# The Institutional Worker

**Published:** 1912-04-20

**Authors:** 


THE
### INSTITUTIONAL WORKER
**Being a Special Supplement to "The Hospital"**

### Editor's Notices.

**Contributions:**
Contributions should be written, or preferably typed, on one side of the paper only, and all articles sent in are accepted upon the distinct understanding that they are forwarded to The Hospital only.

The Editor cannot undertake to return MSS. not used, but when a stamped directed envelope is enclosed the MSS. may be returned if a special request is made.

Accepted articles and paragraphs of news will be paid for after publication at the scale rate.

**Address:**
To prevent delay all contributions and letters on editorial business must be addressed exclusively to the Editor, "The Hospital," 29 Southampton Street, Strand, London, W.C. It is important that this regulation shall be strictly observed.

**Correspondence:**
Correspondence on all subjects is invited. The name and address of correspondents must be given as a guarantee of good faith, but not necessarily for publication.

**Special Articles:**
Special articles are invited, and questions, inquiries, and paragraphs upon all matters relating to the work, administration, and management of general, special, mental, fever, cottage and convalescent hospitals, sanatoria, homes, institutions, societies and organisations for the treatment or care of the sick, injured and dependants of all classes. Every matter affecting the interests and welfare of the staff of all grades working in these institutions will receive special consideration.

**Administrative Medicine, Research and Items of News:**
Special terms will be paid for approved articles on subjects in this wide field by experts who have made some section of it their special study and interest. We wish to give prominence to every movement tending to advance accuracy of diagnosis, efficiency in treatment, and the development of modern methods to eradicate disease and promote the welfare of the suffering.

**A Bureau of Information:**
We invite inquiries and applications for information and help in providing for that numerous class of sufferers who require special care or house-room which they cannot obtain in their own homes or secure for themselves. We cannot, however, prescribe, or recommend practitioners.

**Books for Review:**
Publishers are particularly requested to send advance proofs of any new books of importance whenever possible, as the Editor has made arrangements to publish immediate reviews on a new plan.

**Photographs, Plans, Blocks, and Illustrations:**
It is requested that wherever possible MSS. may be accompanied by illustrations in any of the above forms. The name of the sender and of the article to which it belongs should be written on the back of each photograph, drawing, or block for purposes of identification.

**Local Papers:**
Newspapers containing reports or news paragraphs should be marked and addressed to the Sub-Editor.

**The Coupon System:**
A coupon will be found at the bottom of the third inside page of the cover each week. This coupon must be attached to every question or inquiry to which an answer is desired in The Hospital.

### Next Week.

The Spring Special Number of The Hospital will be published next week, and will contain many important features of the greatest interest to all associated with or engaged in Charitable, Poor Law, and Municipal Administration and Work. The comprehensive circulation which has been provided for this issue will render it of incomparable value to all who desire to secure the immediate attention of the Administrative and Executive Officials of Institutions of every description throughout the United Kingdom. Those of our readers who may be seeking an appointment should certainly avail themselves of this unique opportunity to bring their requirements prominently to the notice of those directly concerned in the management of Hospitals, Infirmaries, and all kindred establishments. The charge for an announcement in the Appointments Wanted column of *The Institutional Worker* is 1s. for twenty-one words, and an advertisement intended for insertion in the Special Number must reach the Publishing Offices, The Hospital Building, 28-29 Southampton Street, Strand, London, W.C., not later than noon on Wednesday next, the 24th inst. The form printed below is for the convenience of our readers who wish to take advantage of the exceptional facilities which the extensive circulation will afford for reaching the Secretaries, Matrons, and other Officials of Institutions, as well as the Responsible Officers in all departments of the Medical, Health, and Sanitary Services.

Appointments Wanted, 21 words ... 1s. 0d.

| | | |
|---|---|---|
| | | |
| | | |
| | | |
| | | |
| | | |
| | | |
| | | |

### Manager's Notices.

**All letters on business matters must be addressed to The Manager, "The Hospital," 28 and 29 Southampton Street, London, W.C.**

**Subscriptions** may begin at any time, and are payable in advance. Cheques and Post-Office orders should be crossed London County and Westminster Bank, Covent Garden branch, and made payable to the Manager of The Hospital.

Rates of Subscription (payable in advance).

| | | |
|---|---|---|
| **UNITED KINGDOM.** | | |
| Three Months | (including Postage) | 2s. 0d. |
| Six Months | do. | 4s. 0d. |
| Twelve Months | do. | 6s. 6d. |
| **FOREIGN AND COLONIAL.** | | |
| Three Months | (including Postage) | 2s. 6d. |
| Six Months | do. | 5s. 0d. |
| Twelve Months | do. | 8s. 8d. |

**Advertisements:**
To ensure insertion, all advertisements for The Hospital must reach the Manager not later than Wednesday morning in each week. For scale of charges see pages v and 2.

**Remittances:**
**It is especially requested that remittances be made by cheque or Postal Order, and in halfpenny stamps only when the amount is under sixpence.**

### OFFICIAL APPOINTMENTS VACANT.

### Poor-Law Vacancies.

Assistant Charge Nurses (2) (£26), Willesden Guardians. Mr. J. H. Haylor, C. to G., 357 High Road, Brondesbury, N.W. April 23.

Assistant Children's Attendant (£18), Hammersmith Guardians. The C. to G., 206 Goldhawk Road, Shepherd's Bush, W. April 22.

Assistant Cook (£26), Holborn Union. Matron, The Infirmary, Archway Road, Upper Holloway, N.

Assistant Cook (£24), Bermondsey Guardians. Matron of the Workhouse, Ladywell Road, Lewisham, S.E. April 25.

Assistant Female Cook (£25), Greenwich Union. Mr. S. Saw, C. to G., Greenwich. April 21.

Assistant Laundress (10s. weekly), Norwich Guardians. Master of the Workhouse, Norwich. April 22.

Assistant Laundry Superintendent (£24), Hammersmith Guardians. The C. to G., 206 Goldhawk Road, Shepherd's Bush, W. April 29.

Assistant Nurse (£25), Atcham Union. E. P. Everest. C. to G., Atcham Union Offices, St. John's Hill, Shrewsbury. April 22.

Assistant Nurse (£20), Hungerford and Ramsbury Union. Mr. H. D'o. W. Astley, C. to G., Hungerford. April 22.

Assistant Nurse (£22 10s.), Watford Union. Mr. F. Wilson, C. to G., Watford. April 23.

Assistant Nurse (£20), Ruthin Union. Mr. R. H. Roberts, C. to G., Ruthin. April 27.

Assistant Nurse (£25), Bedford Union. Mr. W. Payne, C. to G., Bedford. April 29.

Assistant Nurse (£25), Sudbury Union. Mr. G. L. Andrewes, C. to G., Sudbury, Suffolk. April 29.

Assistant Nurse (£25), Dorking Union. Mr. W. James, C. to G., Dorking. April 30.

Assistant Nurse (£25), Wisbech Union. T. H. Stockdale, C. to G., 2 Ely Place, Wisbech. April 27.

Assistant Nurse (£25), Hitchin Union. A. E. Passingham, C. to G., 5 Bancroft, Hitchin. April 26.

Assistant Nursery Attendant (£21 18s.), St. George-in-the-East Guardians. Mr. R. M. Lochner, C. to G., Raine Street, Old Gravel Lane, E. April 30.

Case-Paper Clerks (2) (£80), Chesterfield Union. Mr. R. F. Hartwright, C. to G., Chesterfield. April 23.

Charge Nurse (£30), Cricklade and Wootton Bassett Union. Mr. H. Bevir, C. to G., Wootton Bassett. April 22.

Charge Nurse (£35), Bedwellty Union. Mr. H. J. C. Shepard, C. to G., Tredegar. April 22.

Charge Nurse (£28), Watford Union. Mr. F. Wilson, C. to G., Watford. April 23.

Charge Nurses (£35 and £30), Banbury Union. Mr. E. L. Fisher, C. to G., Banbury. April 25.

Charge Nurses (2) (£35), Bedford Union. Mr. W. Payne, C. to G., Bedford. April 29.

Children's Attendant (£25), Easington Union. Mr. J. M. Longden, C. to G., Easington, Durham. April 25.

Engineer (35s. weekly), Bermondsey Guardians. Mr. E. Pitts Fenton, C. to G., 283 Tooley Street, S.E. April 24.

Female Attendant (£22), Mile End Guardians. Mr. B. Catmur, C. to G., Bancroft Road, Mile End, E. April 25.

Female Attendant (12s. weekly), Bermondsey Guardians. Mr. E. Pitts Fenton, C. to G., 283 Tooley Street, S.E. May 6.

Female General Assistant (£24), Witney Union. Mr. N. J. G. Ravenor, C. to G., Witney, Oxon. April 24.

Head Female Imbecile Attendant (£31), West Ham Union. T. Smith, C. to G., Union Road, Leytonstone. May 1.

Head Nurse (£35), Redruth Union. T. Peter. C. to G. Union Offices, Redruth. April 25.

Industrial Trainer (£18), Wantage Union. Mr. E. B. Ormond, C. to G., Wantage. April 22.

Infant Protection Visitor, etc. (£52), Richmond Union. Mr. P. Umney, C. to G., Richmond, Surrey. April 25.

Laundress, etc. (£20), Aylsham Union. Mr. H. J. Gidney, C. to G., Aylsham. April 22.

Laundry Machineman (23s. weekly), Mile End Guardians. Mr. B. Catmur, C. to G., Bancroft Road, Mile End, E. April 25.

Laundryman (30s. weekly), Norwich Guardians. Master of the Workhouse, Norwich. April 22.

Master and Matron (£45 and £30), Wareham and Purbeck Union. Mr. E. S. Clark, C. to G., Wareham. April 22.

Master and Matron (£120 and £60), Hammersmith Guardians. The C. to G., 206 Goldhawk Road, Shepherd's Bush, W. May 2.

Master's General Assistant and Storekeeper (£25), Chelmsford Union. Mr. A. S. Duffield, C. to G., Chelmsford. April 22.

Night Charge Nurse (£34), Willesden Guardians. Mr. J. H. Haylor, C. to G., 357 High Road, Brondesbury, N.W. April 23.

Nurse (£25), Weymouth Union. Mr. H. A. Huxtable, C. to G., Weymouth. April 22.

Nurse (£26), Hastings Union. Mr. A. R. Inskipp, C. to G., Hastings. April 22.

Nurse (£30), Easington Union. Mr. J. M. Longden, C. to G., Easington, Durham. April 25.

Nurse (£30), Henley Union. Mr. A. R. Lloyds, C. to G., Henley-on-Thames.

Nurse (£25), Worksop Union. J. S. Whall, C. to G. 66 Bridge Street, Worksop. April 23.

Nurse (£35), Bootle Union (Cumberland). J. Clark, C. to G., Broughton-in-Furness.

Nurses (2) (£32 10s.), Stockton Union. Mr. F. B. Watson, C. to G., Stockton-on-Tees. April 23.

Nursery Attendant (£20), Mile End Guardians. Mr. B. Catmur, C. to G., Bancroft Road, Mile End, E. April 25.

Porter and Portress (married couple) (£30 and £20), Wandsworth Union. Mr. F. W. Piper, C. to G., St. John's Hill, Wandsworth. April 24.

Probationer (£10), Willesden Guardians. Mr. J. H. Haylor, C. to G., 357 High Road, Brondesbury, N.W. April 23.

Probationer Nurses (£10), Ecclesall Bierlow Union. J. E. Moulding, C. to G., Union Offices, The Edge, Sheffield. April 30.

Probationer Nurse (£10), Tonbridge Union. N. R. Stone, C. to G., 23 Church Road, Tunbridge Wells. May 2.

Relieving Officer (£120), Bedwellty Union. Mr. H. J. C. Shepard, C. to G., Tredegar. April 22.

Superintendent Night Nurse (£35), Burnley Union. Miss Nugent, Supt., Union Infirmary, Burnley.

Superintendent Nurse (£40), Banbury Union. Mr. E. L. Fisher, C. to G., Banbury. April 25.

Superintendent Nurse (£45), Barton-upon-Irwell Union. Mr. J. W. Whitworth, C. to G., Patricroft, Manchester. April 30.

Superintendents of Children's Homes (married couple) (£40 and £30), Southampton Guardians. Mr. A. J. Walden, C. to G., Southampton. April 27.

Tailor and Wife (Foster-Parents) (£30 and £25), Steyning Union. Mr. A. Flowers, C. to G., Shoreham-by-Sea. April 22.

Ward Sister (£32), Birmingham Union. Mr. R. J. Curtis, C. to G., Birmingham.

Ward Sister (£30), Uxbridge Union. C. Woodbridge, C. to G., 38 High Street, Uxbridge. April 27.

Wardmaid (£16), Cuckfield Union. Mr. E. J. Waugh, C. to G., Haywards Heath. April 25.

### Institutional and Administrative Vacancies.

Matron (£45), Alnwick Infirmary. W. Hindmarsh, Hon. Secretary, 32 Bondgate Without, Alnwick. April 30.

Matron (£50), Yeovil District Hospital. F. W. Mayo, Hon. Sec., Swallowcliffe, Yeovil. May 10.

